# High-Intensity Interval Training Restores Glycolipid Metabolism and Mitochondrial Function in Skeletal Muscle of Mice With Type 2 Diabetes

**DOI:** 10.3389/fendo.2020.00561

**Published:** 2020-08-14

**Authors:** Lifang Zheng, Zhijian Rao, Yifan Guo, Peijie Chen, Weihua Xiao

**Affiliations:** ^1^School of Kinesiology, Shanghai University of Sport, Shanghai, China; ^2^College of Physical Education, Shanghai Normal University, Shanghai, China

**Keywords:** high-intensity interval training, skeletal muscle, type 2 diabetes, glycolipid metabolism, mitochondrial dynamics

## Abstract

High-intensity interval training has been reported to lower fasting blood glucose and improve insulin resistance of type 2 diabetes without clear underlying mechanisms. The purpose of this study was to investigate the effect of high-intensity interval training on the glycolipid metabolism and mitochondrial dynamics in skeletal muscle of high-fat diet (HFD) and one-time 100 mg/kg streptozocin intraperitoneal injection-induced type 2 diabetes mellitus (T2DM) mice. Our results confirmed that high-intensity interval training reduced the body weight, fat mass, fasting blood glucose, and serum insulin of the T2DM mice. High-intensity interval training also improved glucose tolerance and insulin tolerance of the T2DM mice. Moreover, we found that high-intensity interval training also decreased lipid accumulation and increased glycogen synthesis in skeletal muscle of the T2DM mice. Ultrastructural analysis of the mitochondria showed that mitochondrial morphology and quantity were improved after 8 weeks of high-intensity interval training. Western blot analysis showed that the expression of mitochondrial biosynthesis related proteins and mitochondrial dynamics related proteins in high-intensity interval trained mice in skeletal muscle were enhanced. Taken together, these data suggest high-intensity interval training improved fasting blood glucose and glucose homeostasis possibly by ameliorating glycolipid metabolism and mitochondrial dynamics in skeletal muscle of the T2DM mice.

## Introduction

Diabetes is a metabolic disorder that is characterized by hyperglycemia and is due to defects in insulin secretion and/or insulin resistance (IR) (1). The International Diabetes Federation (IDF) estimated that 463 million people (aged 20–79 years) had diabetes mellitus globally in 2019. This estimate is projected to increase to 700 million by 2045 [International Diabetes (2)]. Type 2 diabetes mellitus (T2DM) accounts for more than 90% of patients with diabetes and is characterized by insulin resistance (3). T2DM is recognized as one of the causes of increased mortality and disability, and it also leads to complications such as cardiovascular disease (CVD), neuropathy, retinopathy, and kidney disease (4).

In the human body, skeletal muscle is the largest organ, accounting for ~40–50% of the total body mass. In addition, skeletal muscle is the main target organ that consumes glucose, and it can take in ~80% of glucose by insulin stimulation (5, 6). However, in T2DM, insulin sensitivity in skeletal muscle and the whole body is severely damaged and cannot regulate glucose levels. These factors lead to defects in glycolipid metabolism (e.g., lipid synthesis, lipid oxidation, lipid transport, glycogen synthesis, and glucose transport) and mitochondrial function in skeletal muscle and elevate blood glucose levels (7). Therefore, skeletal muscle is regarded as a potential target organ for the treatment of T2DM. Intramuscular lipids are the energy source for skeletal muscle. However, an increase in fatty acid flow into skeletal muscle or a decrease in fatty acid oxidation causes disordered lipid metabolism, which suppresses insulin-stimulated glucose uptake (8, 9). Mitochondria are the center of glycolipid metabolism and essential organelles for ATP production. It has been suggested that mitochondrial dysfunction is also related to T2DM (10–12). Mitochondrial dysfunction may be caused by several mechanisms such as mitochondrial DNA (mtDNA) mutation, mitochondrial oxidative stress, mitochondrial swelling and disrupted electron potential across the mitochondrial inner membrane (13). In addition, mitochondria are one of the major sites of ROS production in the cell, and mitochondrial dysfunction may increase mitochondrial ROS production. Excessive ROS have been linked to activation of pro-inflammatory molecules, which can disrupt the insulin signaling and contribute to insulin resistance (14).

A vast amount of literature supports a major role of regular exercise in the prevention and treatment of T2DM (15). Traditionally, moderate-intensity continuous training (MICT) has been recommended for patients with type 2 diabetes (16); however, high-intensity interval training (HIIT) is perceived to be more enjoyable, which consists of multiple bouts (30 s−4 min) of near or supramaximal exercise (≥80% of maximal heart rate) separated by periods of active recovery or rest (17). A growing body of evidence suggests that HIIT has a similar or better effect in improving physical fitness and cardiovascular function in patients with type 2 diabetes compared with that of MICT (18, 19). Moreover, studies have shown that acute HIIT reduces blood glucose in patients with type 2 diabetes (20), and 10 weeks of HIIT improves systemic insulin sensitivity of obesity mice (21). Abnormal glucose and lipid metabolism in skeletal muscle are closely associated with T2DM (7). However, the molecular mechanism related to the effect of HIIT on skeletal muscle of individuals with T2DM and obesity has not been fully clarified. Little at al. showed that HIIT improved skeletal muscle mitochondrial content of individuals with IR by regulating the expression of PGC-1α and TFAM (22). HIIT also increased the phosphorylation of IRS (Tyr612), Akt (Ser473), and increased protein content of β-HAD and COX-IV in skeletal muscle of individuals with obesity (23). To date, the effects of HIIT on glucose metabolism, lipid metabolism and mitochondrial dynamics in skeletal muscle of mice with type 2 diabetes have not been investigated. Therefore, the current study aimed to determine the effects of 8 weeks of HIIT on glucose metabolism, lipid metabolism and mitochondrial dynamics in the skeletal muscle of T2DM mice. We hypothesized that HIIT reduces intramyocellular lipids and improves glucose uptake and mitochondrial dynamics in skeletal muscle of T2DM mice.

## Materials and Methods

### Animals

Five-week-old male C57BL/6J mice were purchased from the Model Animal Research Center of Nanjing University (Nan Jing, China). The animals were housed in a room with a light-dark cycle of 12–12 h and temperature of 21 ± 2°C and received water and food *ad libitum*. Following acclimatization to the local environment for 7 days, the mice were randomly divided into two groups: the control diet group (group CON, *n* = 11) and the high-fat diet group (group HFD). The mice in the CON group were fed a chow diet (Research Diet, D12450J; 3.85kcal/g, 10% kcal from fat, 20% kcal from protein, SYSE Ltd., Jiangsu, China). The mice in the HFD group were fed a high-fat diet (Research Diet, D12492; 60% kcal from fat, 20% kcal from protein, 5.24 kcal/g, SYSE Ltd., Jiangsu, China) for 12 weeks. The body weight was recorded weekly. All experimental protocols were approved by the Ethics Review Committee for Animal Experimentation of Shanghai University of Sports (Approval no. 2016006).

### Induction of Type 2 Diabetes Mellitus

The design of the protocol came from previous reports (24) and was amended slightly. Diabetes was induced in mice that were fed a high-fat diet by a single intraperitoneal injection of streptozotocin (STZ, Sigma-Aldrich; Merck KGaA, Darmstadt, Germany) dissolved in citrate buffer (pH 4.4) at a dose of 100 mg/kg, while the control mice received the same volume of citrate buffer (24). Seven days after STZ injection, fasting blood glucose, glucose tolerance and insulin tolerance were measured by blood sampling from the tail vein using a glucometer (Roche), and mice with a fasting blood glucose concentration >13.8 mmol/l were considered diabetic mice (25 of 30) (25). Then, the HFD/STZ induced T2DM mice were randomly assigned to two groups: a T2DM group without exercise (T2DM-SED, *n* = 11) and a T2DM group with high-intensity interval training (T2DM-HIIT group, *n* = 11).

### Exercise Protocol

All mice in the T2DM-HIIT group performed the exercise training program on a mouse treadmill at 25° inclination five times a week for 8 weeks, as described previously with little modification (26). The mice in the T2DM-HIIT group started with a warm-up at 5 m/min for 10 min, in which the HIIT consisted of 10 rounds of 4 min of high-intensity treadmill running interspersed with 2 min of complete rest. The pace during the HIIT increased gradually from 16 to 26 m/min over 8 weeks. The mice in T2DM-SED and T2DM-HIIT groups were kept on a high-fat diet during the 8 weeks of exercise and the calorie intake was recorded every day.

### Glucose Tolerance Tests (GTTs) and Insulin Tolerance Tests (ITTs)

The mice were fasted overnight with free access to drinking water. Following a baseline blood glucose measurement, the blood was taken from the tail at 15, 30, 60, 90, and 120 min following intraperitoneal injection of glucose (1 g/kg body weight). For ITTs, the mice were fasted for 6 h, and insulin was administered by intraperitoneal injection. Blood glucose levels were measured via a drop of tail blood on a glucometer at 15, 30, 60, 90, and 120 min after insulin injection (1 IU/kg body weight) (27). The area under the curves (AUCs) for GTT and ITT were calculated by using Graphpad Prism. To normalize for differences in basal glucose concentrations, these data are displayed as the AUCs with the subtraction of basal glucose concentrations.

### Body Composition

Total fat and lean mass were assessed using Echo MRI (Echo Medical Systems, Houston, TX) at the end of the experiment.

### Tissue Collection and Handling

The mice were sacrificed 36 h after the last exercise session and following a 12-h fasting. The gastrocnemius muscles were obtained immediately and snap frozen in liquid N2. After then gastrocnemius muscles were stored at −80°C until analysis. Blood samples were collected from the mice after 12 h fasting, and serum was separated by centrifugation (3,000 rpm, 15 min) and stored at 80°C until analysis.

### Oil Red O Staining

The gastrocnemius (*n* = 3, three gastrocnemius from three mice in each group) were frozen in optimal cutting temperature compound (OCT) and then cryostat-sectioned at a thickness of 6 μm onto poly-1-lysine slides for lipid deposition analysis by Oil Red O staining using a previously described method (28). The slides were viewed under a bright-field microscope at a magnification of ×200, and images were captured for each muscle section with a Labophot-2 microscope (Nikon Corporation, Tokyo, Japan). The ratio of the red area to the total cross-sectional area of the muscle was calculated to estimate the extent of lipid deposition using Image J (NIH Image, Bethesda, MD) (29).

### PAS Staining

The slides for PAS staining came from the same OCT blocks used for the Oil Red O staining. The slides were stained with periodic acid-Schiff (PAS) according to the manufacturer's protocol (Servicebio, Inc.) to detect glycogen and provide histological details of the muscle structure. The slides were placed in distilled water and treated with periodic acid for 15 min, rinsed well in distilled water, covered with Schiff's reagent for 30 min, and washed in running tap water for 5–10 min. The slides were stained with Mayer for ~30 s and washed in running tap water. The slides were counterstained with hematoxylin for 15 s and washed in tap water. The slides were rinsed in increasing concentrations of alcohol (70, 80, 95, and 100%). Following PAS staining, images were captured for each muscle section under a bright-field microscope (magnification, ×400; Labophot-2; Nikon Corporation). The purple areas were quantified by Image J (NIH Image, Bethesda, MD).

### Transmission Electron Microscopy

Gastrocnemius were immediately fixed in 2.5% glutaraldehyde and post-fixed in 1% osmium tetroxide. After ethanol gradient dehydration, the samples were embedded in Epon 812 (SPI-90529-77-4, Servicebio, Inc.). Ultrathin sections (50–70 nm) were cut and stained with toluidine blue dye. Then, the sections were observed under a transmission electron microscope (hitachi-HT7700; Hitachi Ltd., Tokyo, Japan). The number of mitochondria was counted. A total of six different fields of view (magnification, x5000) were randomly selected from each section.

### Serum Parameters Measurement

Fasting serum insulin levels were determined by using a mouse INS ELISA kit (CEA448Mu; Cloud-Clone Corp., Houston, TX, USA). Total serum cholesterol (T-Chol), triglycerides (TG), high-density lipoprotein (HDL-C), and low-density lipoprotein (LDL) (all purchased from Nanjing Jiancheng Bioengineering Institute, Jiangsu, China) were measured by an automatic biochemical analyzer.

### Detection of Mitochondrial DNA Copy Number by Real-Time PCR

DNA was extracted from the gastrocnemius muscle using a TIANamp Genomic DNA Kit (DP304, Tiangen Biotech Co., Ltd., Beijing, China) in accordance with the manufacturer's instructions. The concentration of the extracted DNA was measured at 260 nm by a microplate reader (BioTek, BioTek Corporation, Vermont, USA). The mitochondrial DNA (mtDNA) copy number was evaluated by determining the ratio of cytochrome b DNA to 18S rRNA and was quantified by real-time qPCR. The qPCR reaction system included SYBR Green (Vazyme, Nanjing, China), nuclease-free water, forward and reverse primers (designed and synthesized by Shanghai Shenggong Biology Engineering Technology Service, Ltd.) and DNA, made to a total volume of 20 μl/well. The StepOne Plus (Applied Biosystems, Carlsbad, USA) was used for amplification by applying the following parameters: denaturation for 5 min at 95°C, 40 cycles of priming at 95°C for 10 s, and annealing at 60°C for 30 s. For cytochrome b, the forward primer was 5′- ATTCCTTCATGTCGGACGAG-3′, and the reverse primer was 5′-AGAAGCCCCCTCAAATTCAT-3′. For 18S rRNA, the forward primer was 5′-TCATAAGCTTGCGTTGATTA-3′, and the reverse primer was 5′-TAGTCAAGTTCGACCGTCTT-3′. Relative gene expression was calculated and quantified with the 2^−ΔΔCt^ method after normalization to the expression level of 18S rRNA.

### Western Blotting

Total proteins were extracted from mouse gastrocnemius muscle using RIPA buffer supplemented with 1 mmol/L PMSF (ST506, Beyotime Institute of Biotech, Jiangsu, China) and a protease and phosphatase inhibitor mixture (P1050, Beyotime Institute of Biotech, Jiangsu, China) (30). The total protein concentration was determined with an enhanced BCA protein assay kit (P0010S, Beyotime Institute of Biotech, Jiangsu, China). Equal amounts of protein from each sample were separated by 10% SDS-PAGE and subsequently transferred to PVDF membranes (Immobilon-P; Millipore, Bedford, MA, USA). After blocking with 5% non-fat dry milk in TBS (containing 0.1% Tween-20). The membranes were incubated with antibodies against acetyl coenzyme A carboxylase (ACC, ab45174, Abcam, Cambridge, United Kingdom, 1:1,000), HMG-CoA reductase (HMGCR, ab174830, Abcam, Cambridge, United Kingdom, 1:1,000), carnitine palmitoyl transferase-1 alpha (CPT-1α, 15184-1-AP, Proteintech, USA, 1:1,000), fatty acid translocase FAT/CD36 (AF2519, R&D, MN, United States, 1:1,000), phospho-glycogen synthase (phospho-GS Ser640, ab2479, Abcam, Cambridge, United Kingdom, 1:1,000), glycogen synthase (GS, ab40810, Abcam, Cambridge, United Kingdom, 1:1,000), glucose transporter 4 (GLUT4, #2213, CST, Beverly, MA, United States, 1:1,000), peroxisome proliferator-activated receptor-g-coactivator-1 alpha (PGC-1α, 66369-1-Ig, Proteintech, USA, 1:500), dynamic-related protein 1 (DRP1, 8570, CST, Beverly, MA, United States, 1:1,000), mitofusin-2 (MFN2, #9482, CST, Beverly, MA, United States, 1:1,000), fission 1 (FIS1, 10956-1-AP, Proteintech, 1:500) and α-tubulin (11224-1-AP, Proteintech, USA, 1:1,000). The appropriate HRP-conjugated secondary antibodies (CST, Beverly, MA, United States) were used to combinate with primary antibodies and the proteins were visualized with enhanced chemiluminescence. α-tubulin was used as a loading control. The bands were visualized with chemiluminescence and quantified by densitometry.

### Statistical Analysis

All data were analyzed using SPSS 23.0 software (IBM, New York, NY, United States) and are presented as the mean ± standard error of the mean (SEM). Statistical analysis was carried out by using one-way analysis of variance, and *post hoc* multiple comparisons were performed using the Bonferroni test. Unpaired Student's *t* tests were used throughout this study to compare two distinct groups using SPSS 23.0 software. A value of *P* < 0.05 indicated statistically significant differences.

## Results

### HFD/STZ Induce the Change of Metabolic Indexes in C57BL/6 Mice

**Figure 1:**
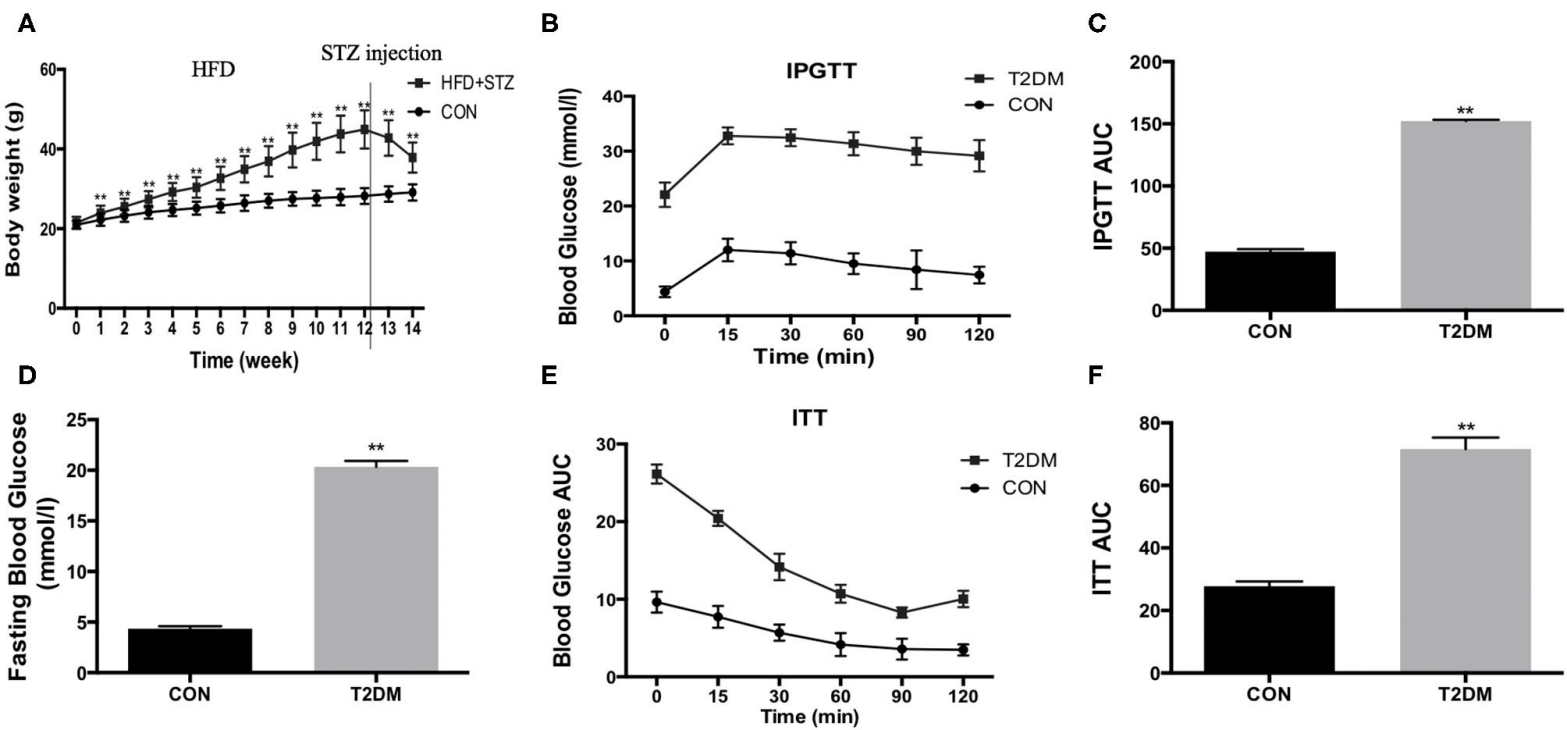
HFD/STZ induce the change of metabolic indexes in C57BL/6 mice. **(A)** Body weight, **(B)** Plots for glucose tolerance tests (IPGTT, 1 g/kg BW) in overnight fasted mice, **(C)** AUC values confirmed impairment of glucose tolerance in T2DM mice, **(D)** Fasting blood glucose, **(E)** Plots for insulin tolerance tests (ITT, 1IU/kg BW) in mice fasted for 6 h, **(F)** AUC values confirmed impairment of insulin tolerance in T2DM mice. All data are presented as mean ± SEM. ^*^p < 0.05, ^**^p < 0.01.

To establish T2DM mice, the mice accepted a high-fat diet and 100 mg/kg STZ injection. We found that the high-fat diet led to increased body weight during the induction period (Figure 1A). However, the body weight was decreased after STZ injection (Figure 1A). In addition, compared to the CON mice, fasting blood glucose was increased (T2DM: 20.35 ± 0.59 mmol/l vs. CON: 4.35 ± 0.24 mmol/l, *p* < 0.01) (Figure 1D). We also detected the glucose tolerance and insulin tolerance, and the results indicated that glucose tolerance (Figures 1B,C) and insulin tolerance (Figures 1E,F) were impaired after high-fat diet and STZ injection. The data above showed that the model of T2DM mice was established successfully.

### HIIT Improves Body Weight and Body Composition in T2DM Mice

**Figure 2:**
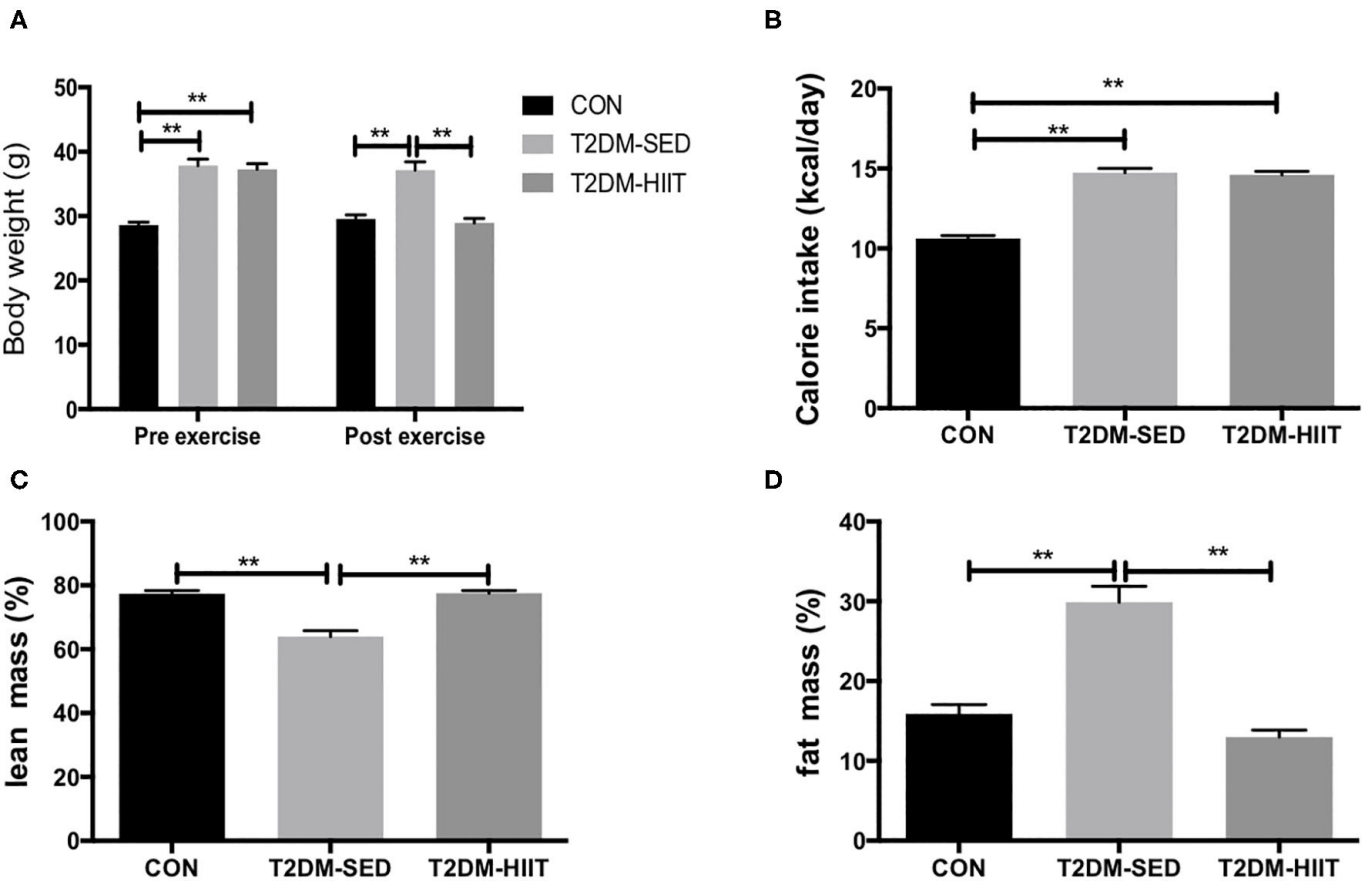
HIIT increases lean mass and decreases fat mass with changing body weight. **(A)** Body weight, **(B)** Calorie intake, **(C)** Percent lean mass, **(D)** Percent fat mass. All data are presented as mean ± SEM. *n* = 11 per group; ^*^p < 0.05, ^**^p < 0.01.

Before exercise, both the T2DM-SED and T2DM-HIIT mice showed significantly higher body weight than those of the CON mice (T2DM-SED: 37.9 ± 1.0 g, T2DM-HIIT: 37.3 ± 0.9 g vs. CON: 28.6 ± 0.5 g, *p* < 0.01) (Figure 2A). After 8 weeks of HIIT, the body weight was significantly decreased in the T2DM-HIIT mice compared to those of the T2DM-SED mice (T2DM-HIIT: 28.9 ± 0.7 g vs. T2DM-SED: 37.1 ± 1.2 g, *p* < 0.01) (Figure 2A). The calorie intake of T2DM-SED and T2DM-HIIT mice was higher than that of CON mice (*p* < 0.01) (Figure 2B), however, there was no significant difference in calorie intake between the T2DM-SED and T2DM-HIIT mice (*p* > 0.05) (Figure 2B).

At the end of the experiment, lean mass was decreased in T2DM-SED mice (CON: 77.4 ± 1.0% vs. T2DM-SED: 64.0 ± 1.8%, *p* < 0.01) (Figure 2C) but was increased in the T2DM-HIIT group (T2DM-SED versus T2DM-HIIT: 77.6 ± 0.8%, *p* < 0.01) (Figure 2C). Fat mass was increased (CON: 15.9 ± 1.2 vs. T2DM-SED: 30.0 ± 2.0%, *p* < 0.01) (Figure 2D) in the T2DM-SED mice; however, fat mass in the T2DM-HIIT mice was decreased when compared to that in the T2DM-SED group (HIIT: 13.0 ± 0.9 vs. T2DM, *p* < 0.01) (Figure 2D).

### HIIT Improves Glucose Handling and the Lipid Profile in T2DM Mice

**Figure 3:**
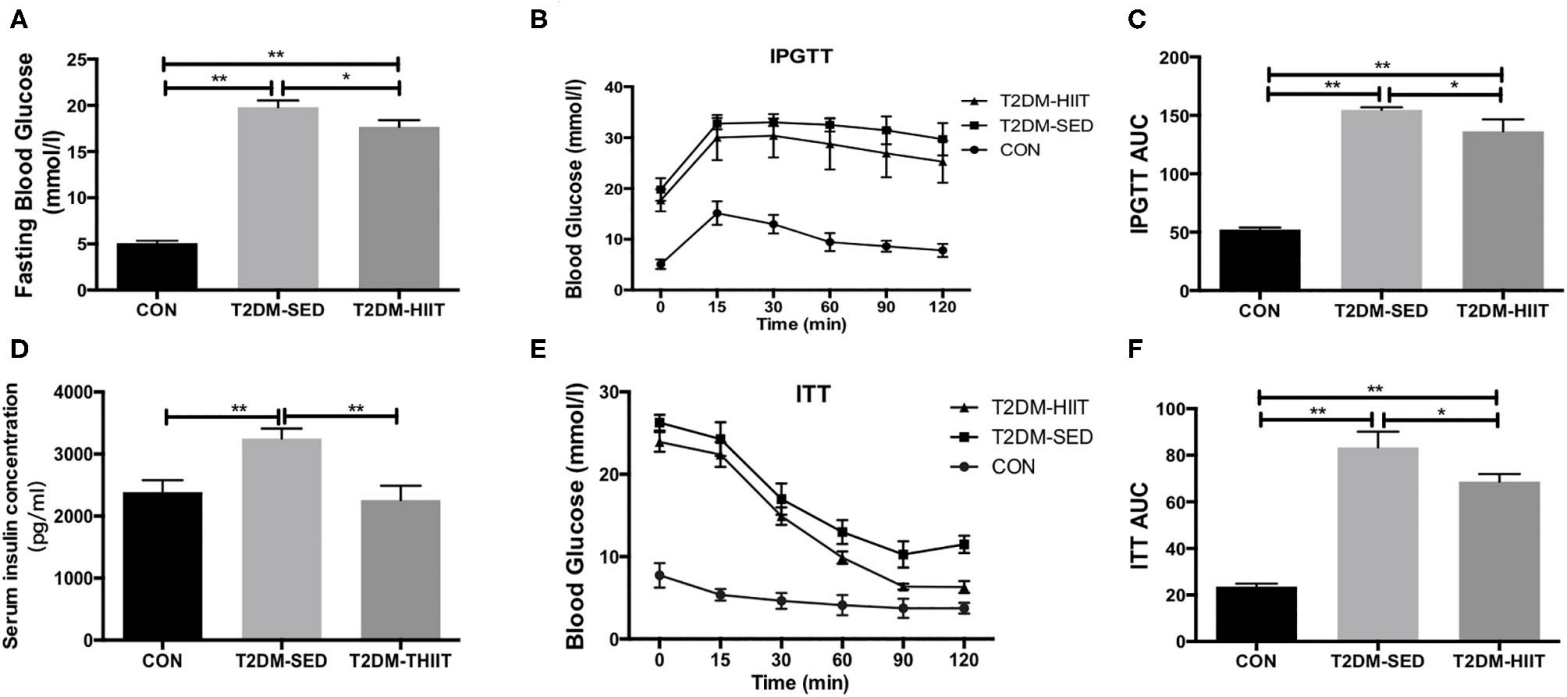
HIIT improves fasting blood glucose, glucose hemostasis and serum insulin in T2DM mice. **(A)** Fasting blood glucose, **(B)** Plots for glucose tolerance tests (IPGTT, 1 g/kg BW) in overnight fasted mice, **(C)** AUC values confirmed improvements of glucose tolerance in T2DM-HIIT group mice, **(D)** Serum insulin concentration, **(E)** Plots for insulin tolerance tests (ITT, 1 IU/kg BW) in mice fasted for 6 h, **(F)** AUC values confirmed improvements of insulin tolerance in T2DM-HIIT group mice. All data are presented as mean ± SEM. *n* = 11 per group; ^*^p < 0.05, ^**^p < 0.01.

The fasting blood glucose was increased in the T2DM-SED mice when compared to that in the CON group (CON: 5.1 ± 0.3 mmol/l vs. T2DM-SED: 19.8 ± 0.7 mmol/l, *p* < 0.01) (Figure 3A), while the fasting blood glucose was decreased in the T2DM-HIIT group (T2DM-HIIT: 17.6 ± 0.7 mmol/l vs. T2DM-SED, *p* < 0.05) (Figure 3A). We also assessed the effect of HIIT on glucose homeostasis and found that the high-intensity interval-trained mice had improved glucose tolerance relative to that of T2DM-SED mice (Figures 3B,C). Similar to the glucose tolerance test, the high-intensity interval-trained mice also had improved insulin tolerance compared to that of T2DM-SED mice (Figures 3E,F).

Serum insulin levels (CON: 2385 ± 193.8 pg/ml vs. T2DM-SED: 3250 ± 159.8 pg/ml, *p* < 0.01) (Figure 3D) and TG (CON: 2.82 ± 0.04 mmol/l vs. T2DM-SED: 3.76 ± 0.13 mmol/l, *p* < 0.01) (Table 1) were increased in T2DM-SED mice compared with those of CON mice but were decreased in the T2DM-HIIT group (T2DM-SED vs. T2DM-HIIT: 2258 ± 229.7 pg/ml; T2DM-SED vs. T2DM-HIIT: 3.21 ± 0.06 mmol/l, *p* < 0.01). TC, HDL-C, and LDL-C levels were not affected by HIIT (*p* > 0.05) (Table 1). An increase in the levels of TC was observed in T2DM-SED mice compared with that of the CON group (*p* < 0.05) (Table 1).

**Table 1:** Effect of HIIT on TG, TC, HDL-C, LDL-C.

	**CON**	**T2DM-SED**	**T2DM-HIIT**
TG(mmol/1)	2.82 ± 0.04	3.76 ± 0.13^**^	3.2 ± 0.06^††^^#^
TC(mmol/1)	5.07 ± 0.18	5.83 ± 0.17^*^	5.78 ± 0.14
HDL-C(mmol/1)	1.26 ± 0.08	1.55 ± 0.12	1.43 ± 0.07
LDL-C(mmol/1)	2.04 ± 0.1	2.36 ± 0.11	2.5 ± 0.03

*TG, triglyceride; TC, total cholesterol; HDL-C, high density lipoprotein; LDL-C, low density lipoprotein*.

* *p < 0.05*,

** *p < 0.01 vs. CON*,

†† *p < 0.01 vs. T2DM-SED*,

# *p < 0.05 vs. CON. All data are presented as mean ± SEM. n = 11 per group*.

### HIIT Reduces Lipid Accumulation and Increases Glycogen Abundance in Skeletal Muscle

**Figure 4:**
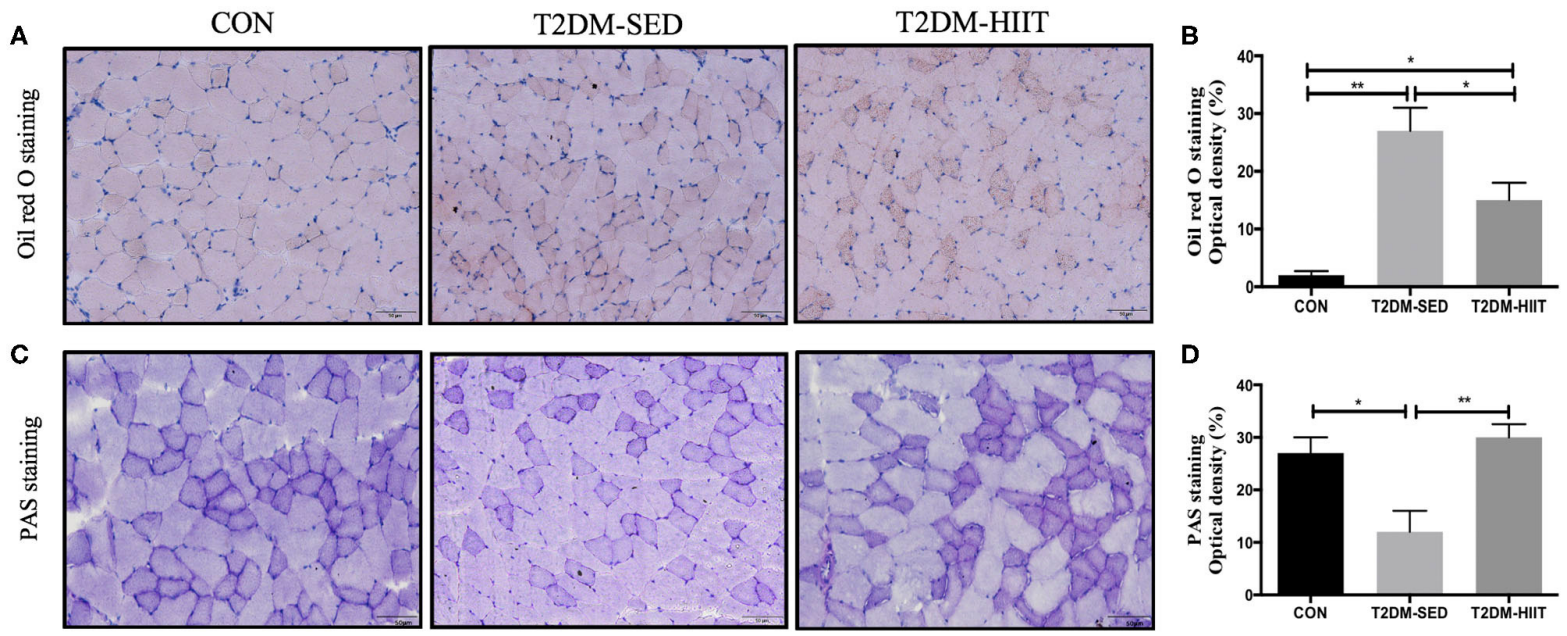
HIIT reduces lipid accumulation and increase glycogen abundance in skeletal muscle. **(A)** Oil red O staining of skeletal muscle (*n* = 3 for each group), **(B)** Quantification of lipid area, **(C)** PAS staining of skeletal muscle (*n* = 3 for each group), **(D)** Quantification of glycogen content. Scale bars, 50 μm. ^*^p < 0.05, ^**^p < 0.01.

Lipid droplets in skeletal muscle of T2DM-SED mice were increased compared to those in the CON group (*p* < 0.01) (Figures 4A,B). Eight weeks of HIIT decreased lipid accumulation in skeletal muscle (frozen sections were stained with Oil Red O, *p* < 0.05) (Figures 4A,B). The PAS stain was chosen to highlight glycogen content that formed in skeletal muscle and is dark blue. The results showed that glycogen content in skeletal muscle of T2DM-SED mice decreased when compared to that of the CON group (*p* < 0.05) (Figures 4C,D). HIIT increased glycogen content in skeletal muscle (*p* < 0.01) (Figures 4C,D).

### HIIT Partially Restores Mitochondrial Morphology and Density in Skeletal Muscle of T2DM Mice

**Figure 5:**
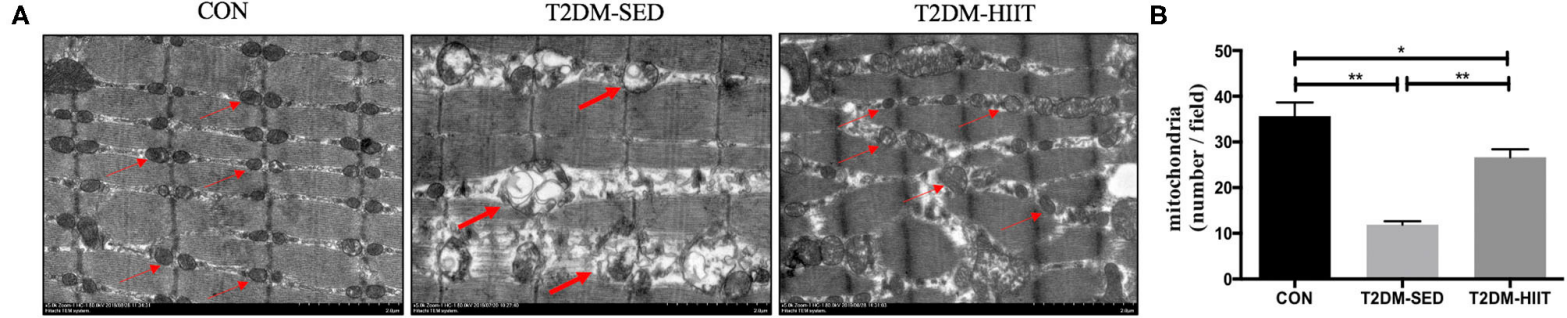
HIIT improves the mitochondrial morphology and density of skeletal muscle in T2DM mice. **(A)** Mitochondria morphological features, **(B)** The number of mitochondria. Thin arrows indicate normal mitochondria; bold arrows indicate damaged mitochondria. Scale bars, 2 μm. *n* = 3 per group; ^*^p < 0.05, ^**^p < 0.01.

We assessed both mitochondrial morphology and quantity by electron microscopy. Electron microscopic observation of skeletal muscle mitochondria showed marked morphological changes and revealed the presence of several vacuole-like structures in T2DM-SED mice compared with those of control mice. The ridge of mitochondria in T2DM-SED mice was also broken or had even disappeared (Figure 5A). The ultrastructural analysis also showed that the mitochondrial density was noticeably reduced in the T2DM-SED mice (*p* < 0.01) (Figures 5A,B). HIIT increased the density of mitochondria and improved the morphology of mitochondria (*p* < 0.01) (Figures 5A,B).

### HIIT Improves the Lipid Metabolism of Skeletal Muscle in T2DM Mice

**Figure 6:**
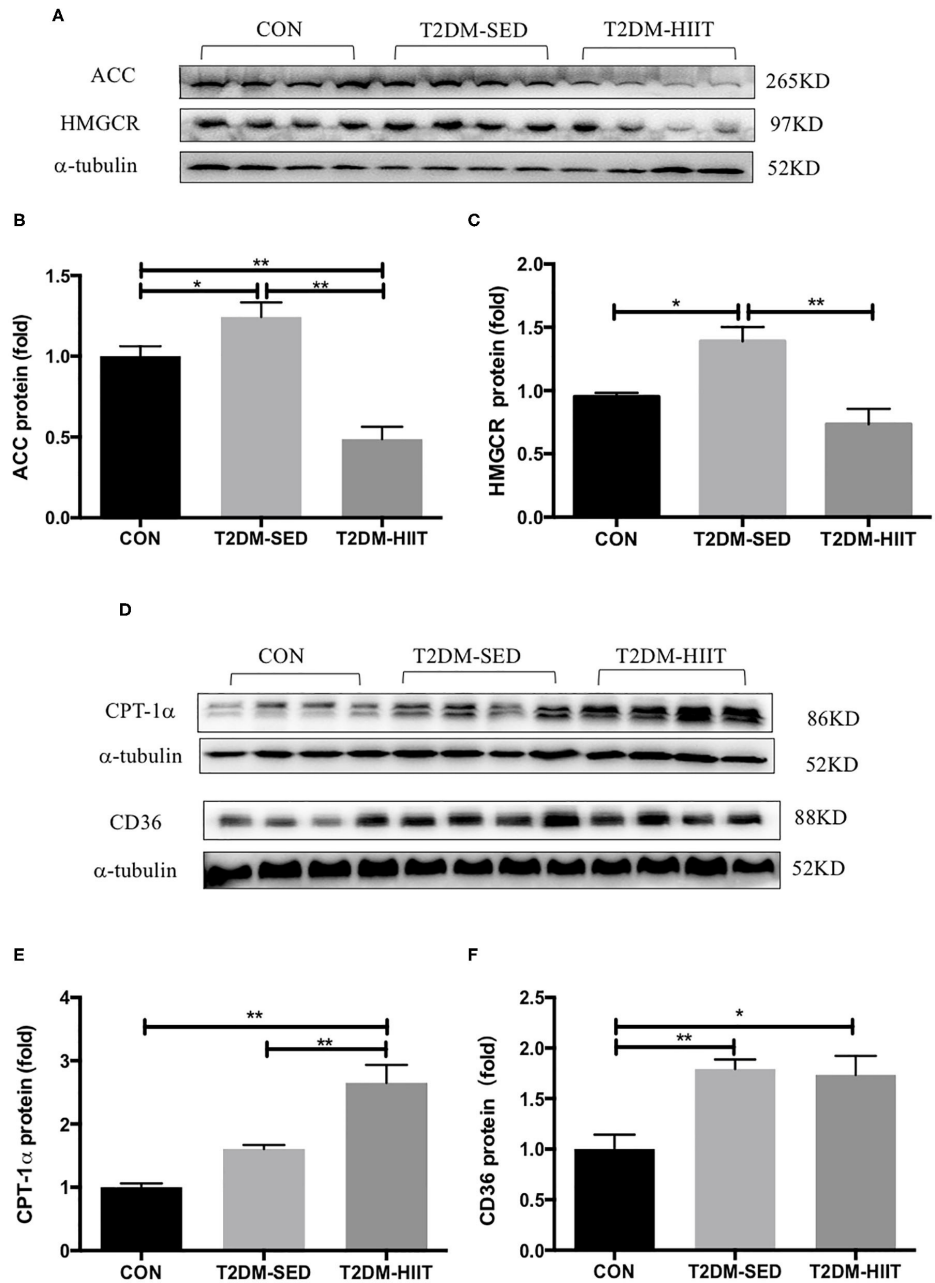
HIIT improves lipid metabolism of skeletal muscle in T2DM mice. **(A,D)** Protein expressions of ACC, HMGCR, CPT-1α, CD36, and internal control α-tubulin in skeletal muscle. **(B,C,E,F)** Quantification of proteins described in **(A,D)** with normalization to protein levels of α-tubulin. All data are presented as mean ± SEM. *n* = 4 per group; ^*^p < 0.05, ^**^p < 0.01.

To elucidate the molecular mechanism by which HIIT reduces lipids, the expression of key proteins related to lipid metabolism in skeletal muscle was investigated. T2DM-SED mice had changes in skeletal muscle protein expression associated with increased lipogenesis compared to those of CON mice, including increments in ACC (1.2-fold, *p* < 0.05) and HMGCR (1.46-fold, *p* < 0.05). HIIT for 8 weeks significantly decreased the protein expression of ACC (0.38-fold, *p* < 0.01) (Figures 6A,B) and HMGCR (0.52-fold, *p* < 0.01) (Figures 6A,C). In addition, CPT-1α and CD36 are involved in the regulation of lipid oxidation and lipid transport. Our results showed that HIIT significantly increased the protein expression of CPT-1α (1.6-fold, *p* < 0.01) (Figures 6D,E), but HIIT had no effect on the expression of CD36 (*p* > 0.05) (Figures 6D,F).

### HIIT Improves the Glucose Metabolism in Skeletal Muscle of T2DM Mice

**Figure 7:**
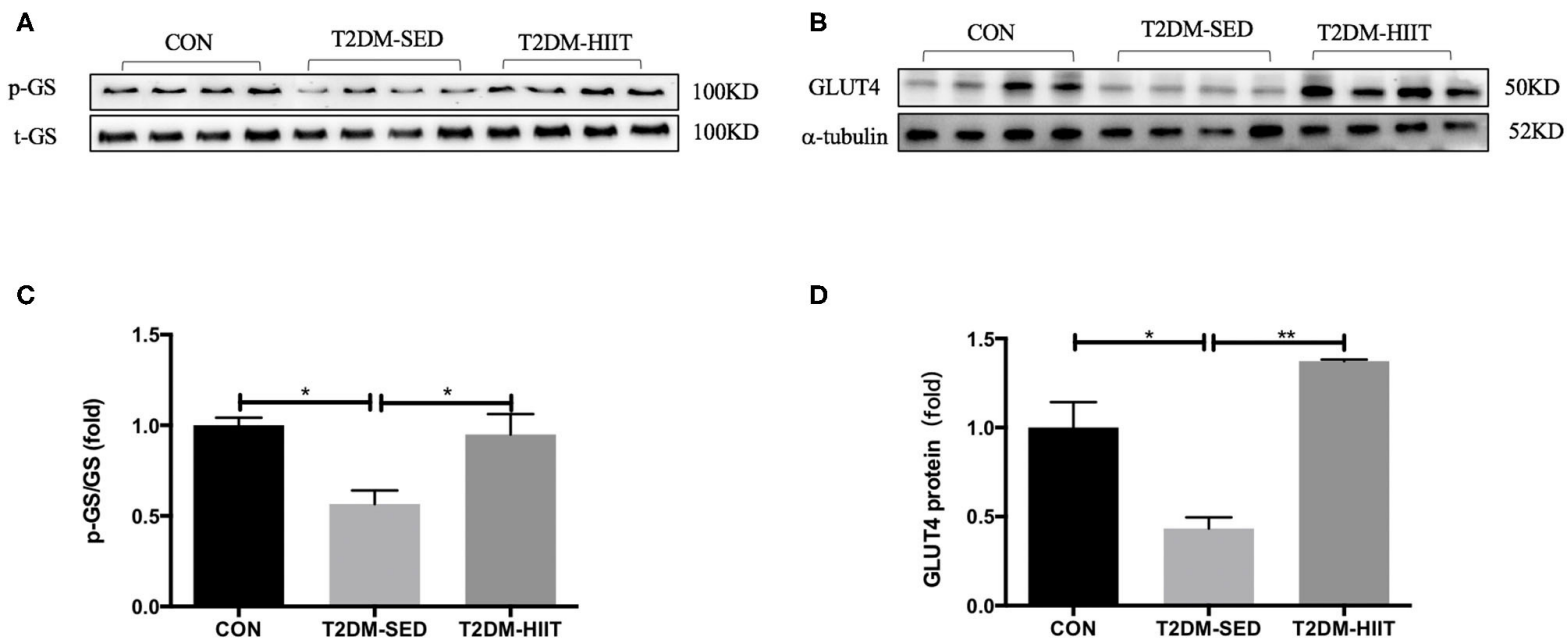
HIIT improves glucose metabolism of skeletal muscle in T2DM mice. **(A,B)** Protein expressions of p-GS/GS, GLUT4, and internal control α-tubulin in skeletal muscle. **(C,D)** Quantification of proteins described in **(A,B)** with normalization to protein levels of α-tubulin. All data are presented as mean ± SEM. *n* = 4 per group; ^*^p < 0.05, ^**^p < 0.01.

Skeletal muscle is one of the key tissues that is responsible for insulin-stimulated glucose consumption. Therefore, the present study measured the protein expression levels of p-GS, GS, and GLUT4. In the T2DM-SED mice, phosphorylation of GS at Ser640 (0.56-fold, *p* < 0.05) (Figures 7A,C) and the protein expression of GLUT4 (0.43-fold, *p* < 0.05) (Figures 7B,D) significantly decreased compared with those of CON mice. while HIIT increased GS serine phosphorylation (1.67-fold, *p* < 0.05) (Figures 7A,C) and GLUT4 protein expression (3.17-fold, *p* < 0.01) (Figures 7B,D).

### HIIT Improves Mitochondrial Biosynthesis and Mitochondrial Dynamics in Skeletal Muscle of T2DM Mice

**Figure 8:**
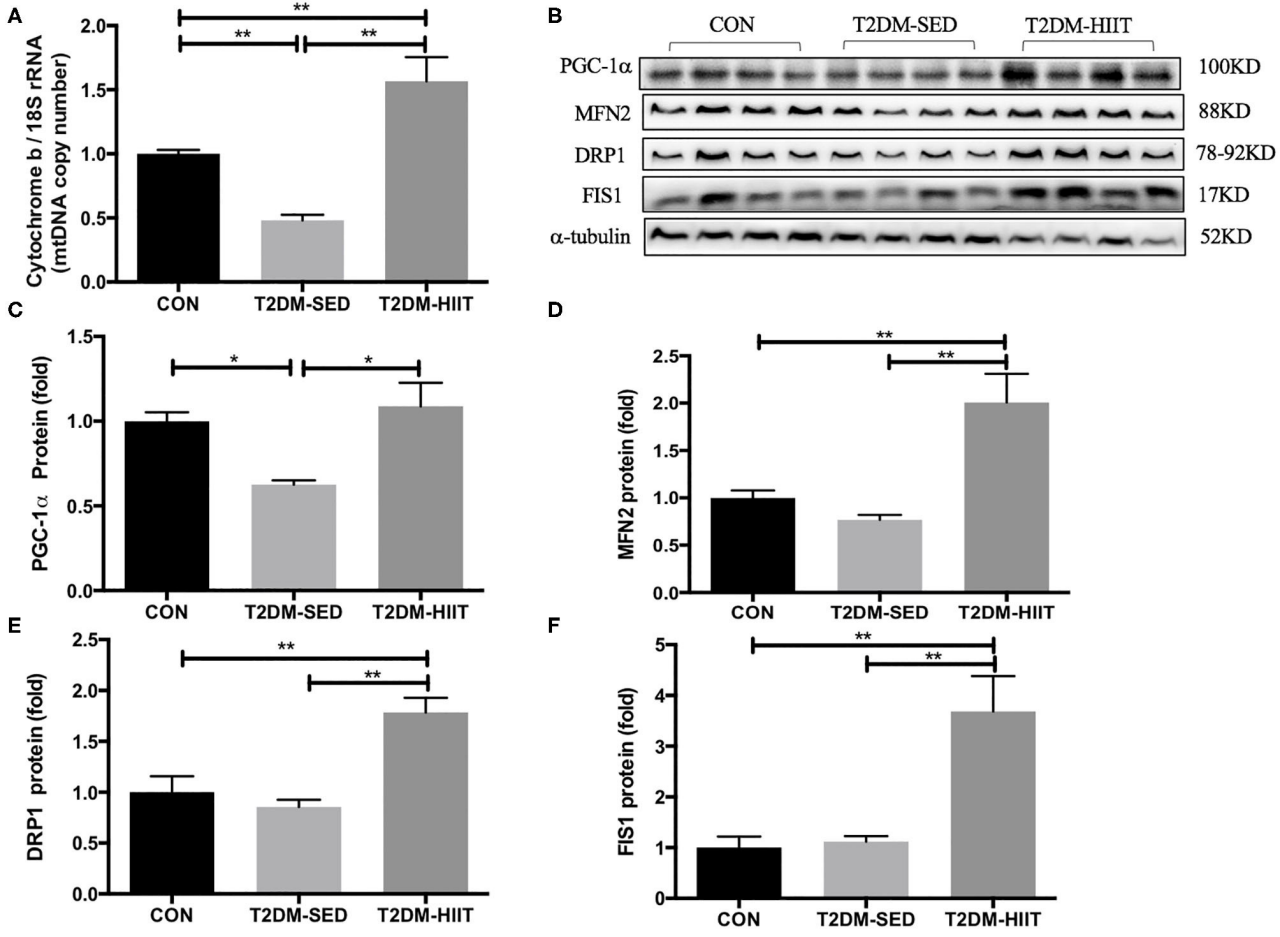
HIIT improves mitochondrial biosynthesis and mitochondrial dynamics of skeletal muscle in T2DM mice. **(A)** Mitochondrial DNA copy number; **(B)** Protein expressions of PGC-1α, MFN2, DRP1, FIS1, and internal control α-tubulin in skeletal muscle. **(C–F)** Quantification of proteins described in **(B)** with normalization to protein levels of α-tubulin. All data are presented as mean ± SEM. *n* = 4 per group; ^*^p < 0.05, ^**^p < 0.01.

Cytochrome b is encoded by mitochondrial DNA, and 18S rRNA is encoded by nuclear DNA (31). The mitochondrial DNA copy number in skeletal muscle was evaluated by determining the ratio of cytochrome b DNA to 18S rRNA. The copy number was lower in the T2DM-SED group than in the CON group (0.48-fold, *p* < 0.01) (Figure 8A), which suggested impaired mitochondrial biogenesis. PGC-1α is a strong regulator of mitochondrial biogenesis. Western blot analysis showed a decrease in PGC-1α protein expression in T2DM-SED mice compared with that of CON mice (0.63-fold, *p* < 0.05) (Figures 8B,C). Moreover, the protein expression of PGC-1α in T2DM-HIIT mice was significantly higher than that in T2DM-SED mice (1.74-fold, *p* < 0.05) (Figures 8B,C).

The expression of proteins that control mitochondrial fusion and fission was analyzed, including the mitochondrial fusion protein MFN2 and mitochondrial fission proteins DRP1 and FIS1. The protein expression of MFN2, DRP1, and FIS1 was not changed in the T2DM-SED group compared with that of CON mice (*p* > 0.05). However, the protein expression of MFN2 (2.61-fold, *p* < 0.01) (Figures 8B,D), DRP1 (2.1-fold, *p* < 0.01) (Figures 8B,E) and FIS1 (3.29-fold, *p* < 0.01) (Figures 8B,F) was dramatically increased in the T2DM-HIIT group.

## Discussion

Mitochondria are necessary organelles for ATP production. Mitochondrial dysfunction is one of the most important mechanisms for T2DM (32), which may be caused by impaired mitochondrial biogenesis and mitochondrial dynamics. Morino et al. found that mitochondrial density was reduced by 38% in IR offspring of T2DM patients (33). In this study, we observed the mitochondrial density with electron microscopy and found that the mitochondrial density in skeletal muscle in T2DM-SED mice also decreased. To study the factors that may lead to a decrease in mitochondria in the skeletal muscle of T2DM mice, we detected the expression of several key regulatory factors of mitochondrial biosynthesis. PGC-1α is a co-transcriptional regulator that is involved in mitochondrial biogenesis. PGC-1α promotes the expression of mitochondrial transcription factor A (Tfam). The interaction between TFAM and mtDNA participates in the regulation of mitochondrial biogenesis (34). Human studies found that the expression level of PGC-1α mRNA in the skeletal muscle of patients with type 2 diabetes was significantly reduced (35, 36). Our results are consistent with these studies, and we found that the mtDNA copy number and the protein expression of PGC-1α were reduced in the gastrocnemius muscle of T2DM-SED mice. These results suggest that PGC-1α and mtDNA are involved in the regulation of mitochondrial biogenesis and are responsible for the reduced skeletal muscle mitochondrial content in T2DM mice. Several studies have shown that exercise induces mitochondrial biosynthesis (37–39). Little at al. showed that HIIT improved skeletal muscle mitochondrial content of individuals with IR by regulating the expression of PGC-1α and TFAM (22). We used different techniques to measure the desired outcomes, including western blotting, electron microscopy, mitochondrial DNA measurements. The results of the present study showed that HIIT significantly increased the mtDNA copy number and the protein expression of PGC-α. The electron microscopy results also showed that the mitochondrial density in skeletal muscle increased after HIIT. Moreover, prior studies have shown that a decrease in mitochondrial content weakened the ability of skeletal muscle to oxidize glucose-derived substrates and led to disordered lipid metabolism and glucose uptake (33, 40). Therefore, our data reveal that HIIT improves lipid metabolism and glucose uptake in T2DM mice, possibly by regulating mitochondrial biosynthesis.

It has been reported that the morphological structure of mitochondria in skeletal muscle of patients with T2DM and their insulin-resistant offspring were altered and contained vacuole-like structures (41), and aberrant mitochondrial morphology is associated with an imbalance in mitochondrial fission and fusion (42). Mfn1/2 regulates the fusion of the mitochondrial outer membrane. DRP1 and FIS1 participate in mitochondrial fission by regulating the mitochondrial outer membrane. Data from the current study found that the protein expression of MFN2, DRP1, and FIS1 was not changed in the skeletal muscle of T2DM-SED mice when compared to CON mice. The electron microscopy results showed that the mitochondria of skeletal muscle in the T2DM-SED mice were impaired. However, studies in humans revealed that the expression of mitochondrial fusion protein was reduced in skeletal muscle of patients with T2DM (42, 43). This difference may be due to the difference in subjects used in the two studies. Therefore, further research is needed to elucidate the changes in mitochondrial dynamics in T2DM mice. Furthermore, high-intensity aerobic exercise and a single bout of aerobic exercise increased the protein and mRNA expression of Mfn1, Mfn2, and FIS1 in the skeletal muscle in both rodents and humans (39, 44, 45). However, few studies reported the effects of HIIT on mitochondrial dynamics in the skeletal muscle of mice with type 2 diabetes. The results of the present study show that HIIT significantly increased the protein expression of Mfn2, Drp1, and FIS1. The electron microscopy results also showed that HIIT improved the mitochondrial morphological structure in skeletal muscle. Several studies have shown that abnormal mitochondrial dynamics are related to glucose metabolism and insulin resistance (46–48). Therefore, the current study suggests that HIIT as a potential treatment to improve mitochondrial dysfunction in T2DM mice. Improved mitochondrial dysfunction may further lead to an improvement in the metabolic state.

Indeed, in the present study, we found that 8 weeks of HIIT decreased fasting blood glucose and serum insulin concentrations in T2DM mice and improved glucose and insulin tolerance. These results are similar to the earlier finding that 8 weeks of HIIT improved glucose tolerance and reduced the systemic glucose and serum insulin concentrations of obese mice (26). Human studies also showed that glucose tolerance was significantly improved in 16 young men following sessions of HIIT, which each involved 4–6 thirty second sprint cycles (49). Dela et al. also found that acute HIIT reduced the blood glucose level of patients with type 2 diabetes (20). Furthermore, Asilah Za'don et al. reported that twenty-five overweight/obese individuals underwent a 12-week HIIT, and HIIT improved insulin sensitivity in obese individuals (50). Moreover, data from the present study revealed that the serum TG in T2DM-HIIT mice was significantly lower than that observed in T2DM-SED mice; however, HIIT did not affect TC, HDL-C, or LDL-C content. In contrast, Wang et al. reported that HIIT lowered the levels of TG, TC and LDL-C in obese mice (26). This difference may be due to differences in the animal models used in the two studies. Therefore, further research is needed to elucidate the effects of HIIT on the serum lipid profile. These results support the current guidelines of the American Diabetes Association (ADA) recommend exercise as an important part of the clinical management of type 2 diabetes (51).

Besides, regular exercise also has been used as an adjuvant therapy to reduce body weight and improve lipid metabolism in obese (52) and subjects with type 2 diabetes (4). Previous studies have shown that 8 weeks of HIIT reduced body weight and fat mass and increased lean mass in obese mice (26). Studies in humans have also revealed that HIIT significantly improved the body weight, fat percentage and BMI of persons with type 2 diabetes (53). In this study, the mice in T2DM-SED and T2DM-HIIT groups were kept on a high-fat diet during the 8-week exercise. Although the body weight of the mice was decreased after STZ injection, the body weight of the T2DM-SED mice has not been changed significantly after the 8 weeks of exercise. However, HIIT exhibited prominent beneficial effects in the reduction of body weight and fat mass, which is consistent with the previous studies (26). Besides, there was no significant difference in calorie intake between the T2DM-SED and T2DM-HIIT mice during the 8-week exercise period. These suggest that HIIT affected either a reduced efficiency in the energy storage or an increased energy expenditure rate or both. Furthermore, the previous studies reported that the reduction of body weight in type 2 diabetes is associated with a fall in fasting blood glucose, a reduction in serum insulin concentrations (54, 55) and improved insulin resistance (56, 57). Our results also showed that fasting blood glucose, serum insulin concentrations, and insulin resistance in T2DM mice were improved after HIIT. These results suggest that the lowered body weight of HIIT may have benefits on improving glucose metabolism and insulin resistance.

We also found an increased glycogen content in the skeletal muscle of the T2DM mice after HIIT. Skeletal muscle is the main target organ that consumes glucose, and it can consume ~80% of glucose by insulin stimulation (5, 6). Glucose uptake and disposal are impaired in skeletal muscle in type 2 diabetic and obese patients (58). GLUT4 is a glucose transporter that is mainly expressed in skeletal muscle, adipose tissue, and myocardium. Insulin promotes glucose uptake of skeletal muscle by promoting the transport of GLUT4 from within the cell to the plasma membrane. In T2DM or insulin resistance, the transport of GLUT4 in skeletal muscle is impaired. A previous study reported that the expression of GLUT4 was decreased in skeletal muscle of T2DM mice (59), and HIIT significantly increased the muscle GLUT4 content (about 2 fold) of db/db mice (60). The results of this study are consistent with a previous study (59, 60) and suggest that HIIT improves glucose insensitivity by upregulating the expression of GLUT4.

Glycogen synthesis in skeletal muscle is directly proportional to the rate of glucose uptake (61). In the case of T2DM or insulin resistance, the insulin-stimulated glucose transport and skeletal muscle uptake glucose are impaired, which affect glycogen synthesis. Carbon-13 nuclear magnetic resonance (13C-NMR) spectroscopy in subjects undergoing hyperglycemic-hyperinsulinemia clamping showed that the glycogen synthase was decreased by 50% or greater in patients with diabetes when compared to healthy individuals (62). The present study also found that the expression of GLUT4 and glycogen content decreased in T2DM-SED mice. Furthermore, exercise could improve skeletal muscle glycogen synthesis by improving insulin resistance and glucose uptake. The results of our study also indicated that insulin sensitivity and glycogen content were enhanced after HIIT. Therefore, the increment of glycogen in muscle after exercise may be related to the increase of insulin sensitivity in skeletal muscle. Moreover, glycogen synthase (GS), a key enzyme in glycogen synthesis, is activated by the allosteric stimulator glucose-6-phosphate (G6P) and by dephosphorylation through inactivation of GS kinase-3 (63, 64). In this study, the phosphorylation of GS was decreased in T2DM-SED mice which may be activated hyperglycemia and low-level glycogen, but the glycogen synthesis was blocked due to skeletal muscle insulin resistance. Sano et al. reported that the glycogen content in skeletal muscle after exercise is lower than that before exercise, and muscle glycogen restored within 24 h post-exercise (65). In the present study, the phosphorylation of GS was increased after HIIT which indicated the inactivation of GS. This may be because the glycogen content had restored when the mice were sacrificed, high-level glycogen suppresses the activation of GS.

Furthermore, excessive lipid accumulation in skeletal muscle is related to obesity and T2DM (66). A number of studies have revealed that ob/ob mice, db/db mice or high-fat diet fed mice have increased lipid accumulation in skeletal muscle (67, 68). Yu et al. (69) indicated that exercise decreased lipid deposition in skeletal muscle of high-fat diet rats. The current study also observed that T2DM-SED mice had increased lipid droplets in skeletal muscle and that 8 weeks of HIIT decreased lipid accumulation in skeletal muscle. At the molecular level, a large number of studies have shown that some genes have a considerable impact on the lipid metabolism of skeletal muscle (70); for example, ACC and HMGCR are endogenous lipogenic enzymes, CPT-1α is involved in the regulation of fatty acid oxidation, and CD36 is a major fatty acid transporter. The results of this study indicate that the expression of proteins related to fatty acid synthesis (e.g., ACC and HMGCR) were reduced, and the expression of proteins related to fatty acid oxidation (e.g., CPT-1α) were enhanced after HIIT. These results suggest that HIIT improves lipid metabolism in skeletal muscle by reducing fatty acid synthesis and increasing fatty acid oxidation. Moreover, CD36 has an important role in the uptake of long-chain fatty acids (71), and long-chain fatty acids are an energy source that could be utilized by skeletal muscle during exercise. Jordy et al. reported that long chain fatty acid uptake is markedly decreased in CD36 knockout mice during contractions/exercise compared to that of WT controls (72). Mice fed a high-fat diet exhibited increased CD36 protein levels in skeletal muscle (73). The present study indicated that the protein expression of CD36 significantly increased in the T2DM mice, and the expression of proteins related to fatty acid oxidation significantly decreased in the skeletal muscle of the T2DM mice. These results suggest dysfunction of mitochondrial lipid oxidation in skeletal muscle of T2DM mice. HIIT increased the protein expression of CPT-1α but had no effect on the protein expression of CD36. Fatty acids may be utilized as an energy source by skeletal muscle during HIIT. Abnormal lipid metabolism contributes to insulin resistance by perturbing insulin signaling pathways (74). Therefore, the results of the current study suggest that HIIT improves glucose homeostasis by regulating the lipid metabolism of skeletal muscle in T2DM mice.

However, there are several limitations to the study, for example, in terms of glucose transport we only measured total GLUT4 content, we did not do the immunohistochemical analysis which would be helpful to see if there is an effect in GLUT4 localization because of exercise in T2DM-HIIT mice. In addition, we only measured the glucose and lipid metabolism by using lipid droplets or glycogen storage suggesting improvements to these metabolic pathways, measures of dynamic glucose and lipid metabolism via indirect calorimetry or high-resolution respirometry would provide greater understanding the improvements in glucose and lipid metabolism of T2DM mice after exercise. The controls for our study are CON mice (no STZ or exercise) and T2DM-SED mice (no exercise), the effects of HIIT in the context of high-fat diet al.one, and the effects of HIIT in the absence of insulin resistance were not included. The inclusion of these groups would further distinguish other effects that are due to hyperinsulinemia/insulin resistance from those that could be a result of the effects induced by the HIIT. Nevertheless, this present investigation suggests that HIIT is an effective strategy to counter the metabolic impairments derived from type 2 diabetes by restoring glycolipid metabolism and mitochondrial function.

In conclusion, we demonstrated that 8 weeks of high-intensity interval training improved fasting blood glucose and glucose homeostasis in T2DM mice by reducing lipid accumulation, increasing glucose uptake, and improving mitochondrial dynamics in skeletal muscle. This deepens our understanding of the mechanism by which high-intensity interval training improves type 2 diabetes.

## Data Availability Statement

The raw data supporting the conclusions of this article will be made available by the authors, without undue reservation.

## Ethics Statement

The animal study was reviewed and approved by the Ethics Review Committee for Animal Experimentation of Shanghai University of Sports (Approval no. 2016006).

## Author Contributions

LZ analyzed the results and drafted the manuscript and performed the western blotting and assisted with histological staining. ZR performed the histological staining. YG helped to keep mouse. PC and WX designed the current study and provided funds. All authors have read and agreed to the final manuscript.

## Conflict of Interest

The authors declare that the research was conducted in the absence of any commercial or financial relationships that could be construed as a potential conflict of interest.
